# Sustainability of collaborative care management for depression in primary care settings with academic affiliations across New York State

**DOI:** 10.1186/s13012-018-0818-6

**Published:** 2018-10-12

**Authors:** Nathalie Moise, Ravi N. Shah, Susan Essock, Amy Jones, Jay Carruthers, Margaret A. Handley, Lauren Peccoralo, Lloyd Sederer

**Affiliations:** 1 Center for Behavioral and Cardiovascular Health, Department of Medicine, Columbia University Medical Center, 622 W. 168th Street, PH9- Room 321, New York, NY 10032 USA; 2 Department of Psychiatry, Columbia University College of Physicians and Surgeons, New York, NY USA; 3 Columbia University Mailman School of Public Health, New York, NY USA; 4 Department of Epidemiology and Biostatistics and Medicine, Center for Vulnerable Populations, University of California, San Francisco, San Francisco, CA USA; 5 Icahn School of Medicine at Mount Sinai, New York, NY USA; 6 Columbia University Mailman School of Public Health, New York, NY USA

**Keywords:** Depression, Sustainability, Primary health care

## Abstract

**Background:**

In a large statewide initiative, New York State implemented collaborative care (CC) from 2012 to 2014 in 32 primary care settings where residents were trained and supported its sustainability through payment reforms implemented in 2015. Twenty-six clinics entered the sustainability phase and six opted out, providing an opportunity to examine factors predicting continued CC participation and fidelity.

**Methods:**

We used descriptive statistics to assess implementation metrics in sustaining vs. opt-out clinics and trends in implementation fidelity 1 and 2 years into the sustainability phase among sustaining clinics. To characterize barriers and facilitators, we conducted 31 semi-structured interviews with psychiatrists, clinic administrators, primary care physicians, and depression care managers (24 at sustaining, 7 at opt-out clinics).

**Results:**

At the end of the implementation phase, clinics opting to continue the program had significantly higher care manager full-time equivalents (FTEs) and achieved greater clinical improvement rates (46% vs. 7.5%, *p* = 0.004) than opt-out clinics. At 1 and 2 years into sustainability, the 26 sustaining clinics had steady rates of depression screening, staffing FTEs and treatment titration rates, significantly higher contacts/patient and improvement rates and fewer enrolled patients/FTE.

During the sustainability phase, opt-out sites reported lower patient caseloads/FTE, psychiatry and care manager FTEs, and physician/psychiatrist CC involvement compared to sustaining clinics. Key barriers to sustainability noted by respondents included *time/resources/personnel* (71% of respondents from sustaining clinics vs. 86% from opt-out), *patient engagement* (67% vs. 43%), and *staff/provider engagement* (50% vs. 43%). Fewer respondents mentioned early implementation barriers such as leadership support, training, finance, and screening/referral logistics. Facilitators included engaging patients (e.g., warm handoffs) (79% vs. 86%) and staff/providers (71% vs. 100%), and hiring personnel (75% vs. 57%), particularly paraprofessionals for administrative tasks (67% vs. 0%).

**Conclusions:**

Clinics that saw early clinical improvement and who invested in staffing FTEs were more likely to elect to enter the sustainability phase. Structural rules (e.g., payment reform) both encouraged participation in the sustainability phase and boosted long-term outcomes. While limited to settings with academic affiliations, these results demonstrate that patient and provider engagement and care manager resources are critical factors to ensuring sustainability.

**Electronic supplementary material:**

The online version of this article (10.1186/s13012-018-0818-6) contains supplementary material, which is available to authorized users.

## Background

More than 100 randomized clinical trials establish that collaborative care (CC) is an effective way manage depression in primary care settings [1–3]. In CC, depression care managers (DCMs), typically nurses or licensed social workers, provide regular, proactive monitoring, treatment-to-target (using standardized screening tools to track progress toward targeted goals through problem-solving therapy and/or working with primary care providers to intensify antidepressants) and registry maintenance (i.e., tracking enrolled patients with depression). CC also includes regular systematic psychiatric caseload reviews and consultation for patients not improving [1, 3–5]. CC has been shown to improve depression, quality of life, and productivity [6–9], while reducing mortality [10] and healthcare costs [11].

Despite randomized control trial findings, penetration of CC remains low. Recent efforts to implement CC in real-world settings, including safety net clinics, suggest multiple implementation barriers [12–15]. The DIAMOND trial, a state-wide Minnesota initiative that included implementation support, training, and monthly bundled payments, found wide variations in implementation strategies [16] but that poor physician and patient participation likely limited program effectiveness [17]. Prior studies identified additional implementation barriers, including lack of resources, space, implementation readiness, leadership support and knowledge, and most importantly, financial barriers such as lack of mental health care reimbursement for care managers and other non-physicians [3, 18–21]. Few prior studies focused on primary care settings where residents are trained; these settings may have unique barriers to implementation and sustainability given variability in provider comfort with and training in depression management [22–24].

In 2012, the New York State (NYS) Office of Mental Health (OMH), in partnership with the NYS Department of Health (DOH), implemented a 2.5-year CC initiative in 32 primary clinics with academic affiliations (where ≥ 1 residents were trained) and where approximately 1 million patients received care. This was a Center for Medicare Services Hospital Medical Home Demonstration project and among the largest statewide initiatives to facilitate CC. To address prior barriers, the CC program provided flexible grant funding for staff and equipment, technical assistance, training, training/upkeep of registries (selected by clinics according to their infrastructure), monthly data submission for monitoring (e.g., clinic screening, enrollment, and remission rates), and quality-improvement activities, as previously described [25]. In 2014, 2 years after implementation, mean depression screening rate across the sites was 85% (vs. 63% in year 1), CC enrollment was 43% (vs. 35%), and clinical improvement (defined as percentage of those enrolled at least 16 weeks with last Patient Health Questionnaire [PHQ] < 10) was 45% (vs. 16%) [25].

After the grant period, OMH and DOH established Medicaid reimbursement rules designed to offer a sustainable financial structure in these 32 clinics and to incentivize high-quality CC implementation statewide. Starting in 2015, they implemented a $150 per-member per-month supplemental payment for adult Medicaid patients receiving depression treatment using the CC model. Clinics received 75% of this fee for enrolling, tracking, and treatment at least once monthly and 25% for achieving continued engagement with the patient and either clinical improvement after 3 months of treatment or documented intervention to address lack of improvement. DOH/OMH supplemented this shift to measurement-based reimbursement (also known as “value-based purchasing” with ongoing training and monitoring. As of 2018, there were over 150 participating clinics, both academic and nonacademic.

To date, there has been little focus on the effectiveness of CC sustainability efforts (e.g., measurement-based reimbursement strategies) or granular assessments of the challenges of transitioning from an implementation into sustainability period. Twenty-six of the original 32 clinics with academic affiliations in the CC demonstration projects opted to enter a sustainability period by enrolling in the Medicaid Reimbursement program (herein called sustaining clinics) and six opted out. This provided an opportunity to examine factors predicting continued CC participation and implementation fidelity. Using a mixed-methods approach, we sought to (1) examine whether clinics sustaining vs. opting out of CC differed in key early implementation fidelity metrics, (2) examine long-term CC fidelity among clinics opting to enroll in a sustainability initiation, and (3) describe barriers and facilitators to CC sustainability. Our aim was to inform future practice, policy, and financing of primary care behavioral interventions.

## Methods

### Overview

**Fig. 1:**
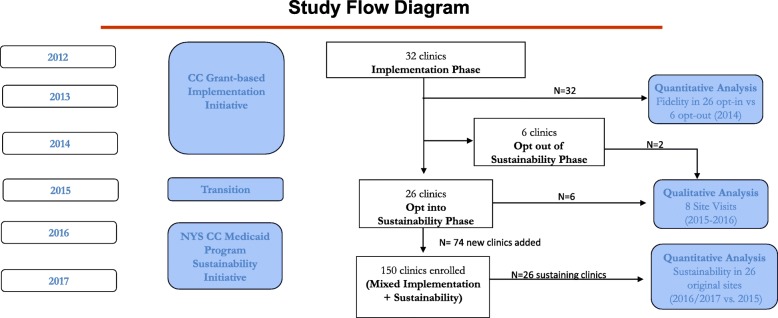
Study flow diagram of the quantitative and qualitative assessments of the NYS Collaborative Care Medicaid Reimbursement Program

Overall, 32 clinics participated in the OMH implementation initiative from 2012 to 2014. In 2015, NYS launched the Medicaid reimbursement program, which included training and measurement-based reimbursement, to support the sustainability phase. Twenty-six of the original 32 clinics opted to enter the sustainability phase while six opted out (Fig. 1). Using descriptive statistics, we compared end of CC implementation metrics in 26 sustaining vs. 6 opt-out clinics. We then analyzed 1- and 2-year trends in CC metrics (newly derived for the sustainability phase) among sustaining clinics. To characterize barriers and facilitators in the sustainability phase, we used purposive sampling methods and conducted 31 semi-structured interviews with psychiatrists, clinic administrators, primary care physicians, and depression care managers (24 at six sustaining clinics, 7 at two opt-out clinics).

### Description of healthcare systems

The 32 clinics represented 19 medical centers with academic affiliations (defined as hospital systems where ≥ 1 residents are trained) caring for approximately one million patients. Overall, 11 healthcare systems (17 clinics) were part of New York City Health and Hospital (H+H), the largest public health care system in the USA, providing care across 70 locations, a low-cost health insurance plan, and population-based care, while 8 (15 clinics) were non-H+H centers (academic health systems only). Clinic-reported populations ranged in size from 2000 patients/year to 30,000 patients/year.

#### Phase 1: Quantitative analyses

We previously published results of the NYS CC Initiative (2012–2014) [25]. Here, we compare sustaining (*n* = 26) and opt-out (*n* = 6) clinics using 2014 metrics: (1) percent and number of patients screened for depression using the Patient Health Questionnaire (PHQ)-2 and/or PHQ-9 in the calendar year; (2) DCM full-time equivalents (FTEs), a proxy for maintaining an integrated, dedicated care manager; (3) number of patients currently enrolled in CC program (including overlap from prior quarters) per DCM FTE; (4) number and percentage of patients screening positive for depressive symptoms enrolled in CC per calendar year; (5) *N* and percentage of individuals enrolled in CC for 16 weeks or greater with PHQ-9 < 10; (6) number and percentage of enrolled individuals with a psychiatric consult; and (7) number and percentage enrolled 6 months still receiving medications or therapy.

We then focused on the sustainability phase metrics (2015–2017) provided quarterly by sustaining clinics (*n* = 26). Measures 1–3 were identical to the implementation period though there may have been reporting variability in “patients enrolled/FTE.” OMH added or refined the remaining as follows: median PHQ-9 of those enrolled; percent of individuals enrolled in CC with ≥ 3 DCM contacts; percent enrolled at least 70 days (10 weeks) with clinical improvement (PHQ-9 < 10 and/or 50% reduction in depressive symptoms); and percent of those in treatment for ≥ 70 days who did not improve but (a) had their treatment changed and (b) received psychiatry consultation. Given the change in metrics, we focused analyses on the change in metrics at 1 and 2 years into sustainability compared to the earliest metrics in the sustainability phase (hereto referred to as baseline).

We used descriptive statistics (counts, medians, interquartile ranges), Mann-Whitney *U* tests, and sign tests to (1) compare implementation metrics in sustaining and opt-out clinics and (2) assess the differences in metrics 1 and 2 years into the sustainability phase compared to baseline sustainability among sustaining clinics. To ensure similarities across analyses (e.g., some measures were year-to-date vs. by quarter), we used the most widely available quarter (quarter 3) for all analyses (including baseline).

#### Phase 2: Qualitative analyses

The OMH Chief Medical Officer invited the 32 original CC initiative clinics to participate in stakeholder interviews about the NYS CC program. Of the original 32 clinics, 30 agreed to participate in site visits (Fig. 1). Using a purposive sampling approach to include both sustaining and opt-out clinics, a psychiatrist (R.S.) and internist (N.M.) not otherwise involved in the CC initiative conducted site visits and interviews between December 2015 and May 2016 to assess barriers and facilitators to sustaining CC. We conducted site visits until we reached saturation (i.e., additional interviews ceased to identify additional themes) [26] in both sustaining and opt-out clinics. Interviewers asked that a PCP (preferably the implementation lead), administrator, DCM, and psychiatrist be available for interview. The New York State Psychiatric Institute institutional review board approved the project.

### Interview guide

We developed the interview guide based on interviews with OMH clinical leadership and prior research on fidelity factors that correlate with CC implementation success [27], including a strong PCP champion, on-site/accessible DCM, perceived financial security, engaged psychiatrist, and warm handoffs (physicians introduce patients to DCMs following a confirmed depression diagnosis). Other measures included number/training/licensure of DCMs, percentage of DCM and psychiatrist time spent in direct patient care, and adherence to weekly multidisciplinary case reviews. We also assessed practice size/setting, patient demographics, number/FTE of residents and attendings, and funding streams. We tailored interviews to interviewees’ positions: DCM, administrator, PCP, and psychiatrist. We asked participants “what makes the program [i.e., OMH’s Medicaid Reimbursement program] challenging,” “what would make it better,” “what are best practices,” and the perceived effectiveness of CC in their clinics. Interviews were semi-structured with open-ended questions (see interview guide in Additional file 1). Information gathered focused on sites themselves, not their larger healthcare systems.

### Qualitative data analysis

We analyzed data pertaining to sustainability barriers and facilitators using NVIVO qualitative research software package, version 11.1. To inform future interventions, we used thematic analysis, incorporating a data-driven inductive approach [28–30]. Two coders (N.M. and R.S.) independently coded all content for meaning and identified central themes [31, 32]. Preliminary codes guided text analysis and as new themes emerged, we assigned inductive codes to data segments [30]. We then compared codes by sustaining and opt-out status. We reconciled disagreements in consensus meetings. Kappa for agreement between coders was 0.84.

## Results

### Quantitative analyses

#### CC implementation metrics in clinics opting to enter the sustainability phase vs. opting out

At the end of the 2-year implementation period, clinics opting to continue the program in the sustainability phase vs. opting out had higher median [interquartile range] care manager FTEs (1.00 [0.75] vs. 0.50 [0], *p* = 0.002) and achieved higher clinical improvement rates (46.0% [53.0] vs. 7.5% [23.0], *p* = 0.004). We found no significant differences (*p* < 0.05) in median rates of depression screening (96.5% [13.0] vs. 87.0% [41.0], *p* = 0.51), enrolled patients per calendar year (43.0% [45.0] vs. 34.0% [13.0], *p* = 0.22), and psychiatric consultations per quarter among enrolled patients (100% [44.0] vs. 90% [100], *p* = 0.53) (Table 1). Differences in clinic census (5669 [7635] vs. 2686 [1829], *p* = 0.06) and enrolled patients/DCM FTE (137.8 [89.0] vs. 58.0 [61.0], *p* = 0.07) approached significance. Among the six opt-out clinics, reasons for opting out of the sustainability phase were “staffing” (*n* = 5) and billing infrastructure (*n* = 1).

**Table 1:** Implementation-end fidelity metrics in sustaining vs. opt-out clinics

Metric (median, [IQR])	Sustaining clinics (*n* = 26)	Opt-out clinics (*n* = 6)	*P* value
Total census at clinic	5669 [7635]	2686 [1829]	0.06
% screened per calendar year^1^	96.5% [13.0]	87.0 [41.0]	0.51
Depression care manager full-time equivalent	1.00 [0.75]	0.50 [0]	*0.002*
Number of participants enrolled/FTE^2^	137.8 [89.0]	58.0 [61.0]	0.07
% of depressed patients in calendar year enrolled into collaborative care program^3^	43.0% [45.0]	34.0% [13.0]	0.22
% currently enrolled in third quarter with psychiatry consultation^4^	100% [44.0]	90% [100]	0.53
% enrolled for 6 months and still on med/therapy (%)^5^	15.0% [21.0]	42.0% [85.0]	0.77
% of patients enrolled in collaborative care ≥ 16 weeks with PHQ9 < 10^6^	46.0% [53.0]	7.5% [23.0]	*0.004*

^1^% unique adult patients per year from the outpatient site who received a PHQ-2 or PHQ-9 over number of patients

^2^Number of patients currently enrolled in collaborative care Quarter 3 per depression care manager Full Time Equivalent

^3^% unique adult patients per year from the outpatient site screening positive for depression who enrolled in physical-behavioral health care coordination program (Collaborative Care Initiative) per year

^4^% of unique adult patients enrolled in the Collaborative Care Initiative for which a psychiatric consultation occurred during this reporting period

^5^% of unique adult patients enrolled in the Collaborative Care Initiative still receiving medication and/or psychotherapy six (6) months after enrollment

^6^% unique patients enrolled in the Collaborative Care Initiative ≥ 16 weeks whose PHQ-9 < 10

*p*<0.05 was considered statistically significant

#### CC metrics 1 and 2 years into sustainability among 26 sustaining clinics

Compared to baseline, at 1 and 2 years into the sustainability phase (median [interquartile range]), sustaining clinics reported stable screening rates (88.5% [19.0] vs. 86.0% [31.0] and 91.0% [18.0]) and DCM FTEs (1.00 [1.00] vs. 1.00 [1.00] and 2.00 [1.00]). They also saw stable median PHQ-9 (10.5 [5.0] vs. 9.75 [6.0] and 10.0 [3.0]) and percent enrolled ≥ 70 days without improvement but with a psychiatry consult or treatment change rates (Table 2). Clinics reported improvements in the proportion of patients enrolled in CC with ≥ 3 DCM contacts (29.0% [33.0] vs. 24.0% [33.0] and 40.5% [24.0]) and of patients enrolled ≥ 70 days and in remission (33.0% [22.0] vs. 49.0% [25.0] and 58.0% [19.0]). However, patient enrollment/FTE significantly decreased (56.0 [36.0] vs. 44.6 [18.0] and 36.5 [37.5]). The number of patients/FTE screening positive for depressive symptoms remained stable in the same period (297 [354] vs. 281 [204] and 337 [294]) (Table 1).

**Table 2:** New York State Collaborative Care Medicaid Program Reporting Metrics (2015–2017): year 1 and year 2 sustainability (vs. baseline) in 26 clinics opting to sustain CC after a 2-year implementation initiative (We used quarter 3 data for each year to ensure comparable results (e.g., some metrics reported for calendar year and others for a given quarter))

Metric (median, [IQR])	Implementation end (2014)^†^	Baseline sustainability (2015)^†^	*P* value (2015 vs. 2014)	Year 1 sustainability (2016)	*P* value (2016 vs. 2015)	Year 2 sustainability (2017)	*P* value (2017 vs. 2015)
% screened	96.5% [13.0]	88.5% [19.0]	*< 0.001*	86.0% [31.0]	0.38	91.0% [18.0]	*0.01* ^††^
Depression care manager full-time equivalent	1.00 [0.75]	1.00 [1.00]]	0.08	1.00 [1.00]	0.79	2.00 [1.00]	0.09^††^
Number of participants enrolled/FTE		56.0 [36.0]	N/A	44.6 [18.0]	0.23	36.5 [37.5]	*0.004*
Number of participants screening positive for depression/FTE		297 [354]	N/A	281 [204]	0.17	337 [294]	0.81
Median Patient Health Questionnaire of current enrollees		10.5 [5.0]	N/A	9.75 [6.0]	0.41	10.0 [3.0]	0.23
% enrolled with ≥ 3 contacts		29.0% [33.0]	N/A	24.0% [33.0]	0.17	40.5% [24.0]	*0.03*
% enrolled 70 days with improvement (PHQ < 10 or 50% reduction)		33.0% [22.0]	N/A	49.0% [25.0]	*0.009*	58.0% [19.0]	*0.004*
% not improved after 70 days with psychiatry consult		55.0% [47.0]	N/A	57.5% [29.0]	1.00	80.5% [49.0]	0.11
% not improved after 70 days with treatment change		48.0 [36.0]	N/A	54.0% [39.0]	1.00	73.0% [53.0]	0.33

^†^Only 3 comparable metrics were available for implementation and the sustainability initiatives. It is unclear whether clinics were reporting enrollment rates similarly between implementation and sustainability phases (calendar year vs. per quarter)

^††^Compared to implementation phase, year 2 sustainability saw significantly higher DCM FTE (*p* = 0.004) and lower screening rates (*p* = 0.03)

*p*<0.05 was considered statistically significant

### Site visit characteristics and sustainability fidelity

Eight site visits yielded 31 semi-structured interviews (7 psychiatrists, 8 clinic administrators, 8 PCPs, and 8 DCMs) (Fig. 1). We conducted 7 interviews at 2 opt-out sites (A and F) and 24 interviews at six sustaining sites; most interview participants (73%) were female (Table 3). Sites were representative of the overall population: at the end of the implementation phase (2014), opt-out (vs. sustaining) sites participating in the qualitative interviews had fewer overall DCM FTEs (both 0.5–1 vs. ≥ 1.0), fewer enrolled patients/DCM FTE (30–103 vs. 70–179) and lower improvement rates (0–23% vs. 14–66% in sustaining clinics).

**Table 3:**
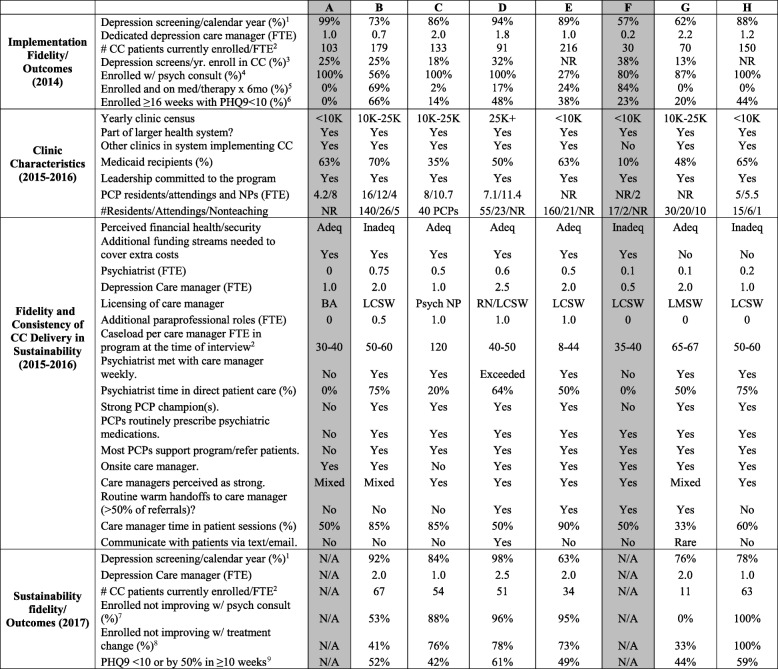
Clinic-level characteristics and implementation-related outcomes of 8 sites participating in the New York State Collaborative Care program in sustainability phase (gray = opt-out clinics)

*LCSW* licensed social worker, *BA* Bachelor of Arts, *Psych NP* psychiatric nurse practitioner, *FTE* full-time equivalent, *PHQ9* Patient Health Questionnaire, *CC* collaborative care, *RN* registered nurse

^1^% unique adult patients per year from the outpatient site who received a PHQ-2 or PHQ-9 over number of patients

^2^Number of patients currently enrolled in collaborative care Q3 per depression care manager FTE (there may have been variations in how clinics reported these from implementation to sustainability).

^3^% unique adult patients per year from the outpatient site screening positive for depression who enrolled in physical-behavioral health care coordination program (Collaborative Care Initiative) per year

^4^% of unique adult patients enrolled in the Collaborative Care Initiative for which a psychiatric consultation occurred during this reporting period.

^5^Number of unique adult patients enrolled in the Collaborative Care Initiative still receiving medication and/or psychotherapy six (6) months after enrollment

^6^% unique patients enrolled in the Collaborative Care Initiative ≥ 16 weeks whose PHQ-9 < 10

^7^% of patients enrolled 70 days and not improved who received a psychiatric consultation during this reporting period

^8^% of patients enrolled 70 days and not improved who received a treatment change during this reporting period

^9^% of patients enrolled 70 days (10 weeks) with PHQ9 < 10 or PHQ9 reduced by 50%

#### Clinic characteristics

Overall, all clinics were part of larger healthcare systems, the majority of which had other clinics implementing CC through the OMH/DOH program. All clinics had more resident (15–160 residents at 4–16 FTEs) than attending (6–26 attendings at 4–11 FTEs) PCPs. Opt-out clinics were slightly smaller clinics (i.e., cared for fewer patients) and had fewer overall PCP FTEs than sustaining clinics. Two participating clinics (B and E) were public healthcare systems within H+H. All but one (opt-out) site cared for a majority Medicaid population.

#### CC metrics and implementation fidelity in the sustainability period

All clinics reported leadership support for CC and five of eight felt that the CC program was financially secure, though six of eight clinics (including both opt-out clinics), were using additional funding streams to cover extra costs in the sustainability phase. Fifty percent of all sites reported routine warm handoffs. All six sustaining sites reporting weekly psychiatry consults and imbedded psychiatrists who spent 20–75% of their time in direct patient care. These sustaining clinics also noted strong PCP champions, PCPs who routinely prescribed psychiatric medications and full support of PCPs for the program. Five of six sustaining sites had onsite DCMs and reported caseloads of ≥ 50 patients/DCM FTE at the time of our interview. Four of six sites reported that care managers time spent > 50% of their time in patient sessions and reported investing additional resources in hiring a paraprofessional to assist the care manager in tasks such as registry upkeep (Table 3).

Meanwhile, both opt-out sites reported low psychiatry FTEs dedicated to the program (0 and 0.1 FTE) and 0% psychiatrist time spent in direct patient care. These opt-out sites reported no weekly psychiatry meetings with care managers, lacked PCP champions and both reported care manager caseloads of < 50 patients/FTE and that care managers spent only 50% of their time in direct patient care. Neither had paraprofessionals and 1 reported that PCPs were not routinely prescribing psychiatric medications (Table 3).

#### Barriers to CC implementation in the sustainability phase (**themes** are bolded, sub-themes are underlined and italicized)

**Fig. 2:**
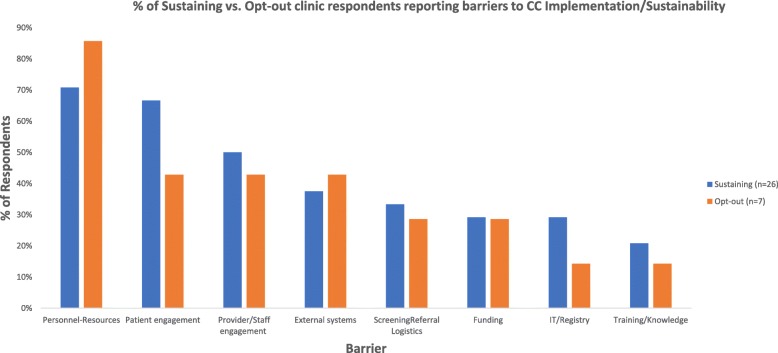
% of Sustaining vs. Opt-out clinic respondents reporting barriers to CC Implementation/Sustainability (*n* = 31 respondents)

**Time-personnel resources** (74% of respondents; 71% at sustaining clinics and 86% at opt-out clinics). Interviewees noted *inadequate number of DCMs* (46% sustaining vs. 71% opt-out) to meet clinical demands as well as *competing DCM roles* (13% sustaining vs. 29% opt-out) in clinics (e.g., registry upkeep). Respondents also described *inadequate MD resources*, though sustaining sties particularly noted *competing PCP demands and time constraints*: “[Providers] are getting hammered with increased number of items they are supposed to manage in the visit. We have to screen every patient for risk of domestic violence…falls” (Sustaining PCP) while opt-out clinics focused on *lack of psychiatry resources* “case consultation with psychiatrist didn’t happen. Psychiatrist available by telephone for consultation, but no set time to have him available. If you are in a crisis to call a psychiatrist this is ok, but overall not” (Opt-out PCP). Other factors included *inadequate space* and *psychosocial resources*, as well as  *inadequate personnel-resources* in general (e.g., some clinics did not adequately plan and think through logistics, personnel to patient ratios, and FTEs) (Table 4; Fig. 2).

**Table 4:** Barriers to Collaborative Care implementation/sustainability 1–2 years after a 2 year-implementation program among clinics opting in vs. out of a Medicaid reimbursement sustainability initiative

Barriers	Total (%)	Sustaining (%)	Opt-out (%)	Quotes
Time-personnel resources	74	71	86	
Inadequate personnel resources	16	13	29	“Did not think about logistics issues, no thought of sites, ratios, right sizing, right timing of this −3.5-4 h of .1 FTE for 6 PCPs” (opt-out site)
Competing DCM roles	16	13	29	“Currently, the administrator of the program (a certified care coordinator who can do problem solving therapy and motivational interviewing) is only funded for .5FTE, so the other 50% of her time is devoted to a diabetes program in the primary care clinic.” (Opt-in PCP)
Inadequate MD resources	26	25	29	“[Providers] are getting hammered with increased number of items they are supposed to manage in the visit. We have to screen every patient for risk of domestic violence…falls…travel to West Africa for Ebola, and unfortunately, this [depression screening] is one more measure” (Opt-in PCP) “Case consultation with psychiatrist did not happen. Psychiatrist available by telephone for consultation, but no set time to have him available. If you are in a crisis to call a psychiatrist this is ok, but overall not” (Opt-out PCP).
Inadequate number of DCMs	52	46	71	“Only have 1 provider. It’s impossible to have her take care of all the depress[ed] people… Appointments are not always given the day of the [PCP] appointment” (opt-in PCP)
Inadequate psychosocial resources	19	21	14	“At the same time, many patients want and/or need more intensive psychiatric treatment than can be offered in this setting (such as a day program or intensive outpatient treatment).” (Opt-in Psych)
Inadequate space	13	13	14	
Patient engagement	61	67	43	
Lack of patient engagement	16	17	14	“No shows [are a problem] because rescheduling with the psychiatrist takes another 60 days” (Opt-in Psych).
Culture- language	16	13	29	“[it was] hard to recruit someone who spoke language, used language lines (had to pull staff to translate)” (Opt-out Admin).
Infeasible warm handoffs	19	21	14	“[we had a] part time care manager who cannot have a warm handoff [which] is much less effective” (opt-in DCM).
Patient nonadherence	39	42	29	“It’s challenging to make sure that patients continue to follow up. It is difficult because it affects the entire person and you have to get them to participate in the plan. Loss to follow up.” (Opt-in PCP)
Stigma	13	13	14	
Provider/staff engagement	48	50	43	
Miscommunication	3	4	0	“But if psychiatrist thinks that the medication needs to be increased, and then have to tell physician this message, which can sometimes be odd. The interaction between psychiatrist and physicians should be improved.” (Opt-in DCM)
Lack of DCM engagement	6	4	14	
Lack of PCP engagement	35	38	29	“doctors are uncomfortable either because they might get more work to do” (Opt-in PCP)
Provider continuity	19	21	14	“DCM turnover with mixed experiences of quality of DCMs” (Opt-in Admin) “Residents are not always engaged because you have to teach them all over again.” (Opt-out DCM)
Psychiatrist engagement	13	13	14	“Psychiatrists not motivated or interested in this model” (Opt-out Psych)
Staff engagement	3	4	0	
External factors	39	38	43	
Healthcare system/guidelines	3	4	0	“We used to consider PHQ < 10 as remission, but now guidelines say it is under 5. PHQ < 5 is probably not realistic.” (Opt-in PCP)
Competing primary care initiatives	6	4	14	“PMDs are getting hammered with increased number of items they are supposed to manage in the visit. We have to screen every patient for risk of domestic violence, risk of falls, etc. We’d love to screen patients for substance use” (Opt-in PCP)
Other	39	38	43	*Mental health infrastructure:* “the sickest patients psychiatrically have to be referred out. That coordination is tough as it is difficult to collaborate with mental health providers outside of the clinic.” (Opt-in PCP). *Restrictive CC initiative enrollment “*Patients who do not have Medicaid are still screened, so they get “light touches” with the DCM to help get them referred into therapy.” (Opt-in Admin)
Screening/referral	32	33	29	
Complicated screening/referral logistics	3	0	14	“Screening was initially tough given cultural barriers among patients and staff as well as tester fatigue” (Opt-in Admin). “[the] MA [medical assistants] did PHQ2 but hard to make sure to alert the doctor about PHQ2 and to do the PHQ 9 if positive” (opt-out PCP).
Triaging patients	29	33	14	
Funding	29	29	29	
Complex funding stream	26	25	29	“For patients with commercial insurance, each insurance has a different requirement/payment structure” (opt-out admin)
Insufficient funding	13	13	14	“Billing is not enough, but it’s close. If CM’s have between 70–80 patients that they bill for consistently might break even. Cannot bill retainable for everyone. Does not cover psychiatry/PCP coordinator” (Opt-in PCP).
Information technology (IT)/Registry	26	29	14	
Paper referral-screening/EHR	3	4	0	
Registry management	23	25	14	“Registry has been very challenging because there is nothing automated about the registry and the amount of work to feed into the day.” (Opt-in Admin)
Training/knowledge	19	21	14	
Inadequate DCM training	3	4	0	
Inadequate physician knowledge	16	17	14	“Educating the PCPs, getting them more involved, have them be less afraid of prescribing and increasing the dose. They cannot see patients every month because they are so busy.” (Opt-in Psych)
Lack of buy-in/implementation readiness	10	13	0	“There is too much orthodoxy, so this would be better if there were more flexibility. If the outcomes are coming out well, why do you have to replicate the studies that were done?” (Opt-in Admin)

*Admin* clinic administrator, *DCM* depression care manager, *Psych* clinic CC psychiatrist, *PCP* primary care provider/champion

**Patient engagement** (61% of respondents; 67% sustaining and 43% opt-out) was another major barrier to CC sustainability, corresponding to quantitative findings of decreasing enrollment rates, particularly relevant to sustaining clinics attempting to sustain CC and bill for patients through the program. Respondents described high no-show rates and poor completion of problem-solving therapy homework (*patient non-adherence*; 42% sustaining and 29% opt-out) which contributed to long-waiting lists and delayed care. Respondents also described reluctance to enter treatment due to *stigma* as well as poor health literacy and cultural misconceptions of depression and its treatment (*culture-language*; 13% sustaining vs. 29% opt-out). Language barriers also made it complicated to hire DCMs: “[it was] hard to recruit someone who spoke language, used language lines (had to pull staff to translate)” (Opt-out Admin). Patient engagement closely related to *infeasible warm handoffs* (19% of respondents): “[we had a] part time care manager who can’t have [time for] a warm handoff [which] is much less effective” (Sustaining DCM).

**Provider/staff engagement** (i.e., staff, PCP, DCM, and psychiatrist engagement and communication as well as provider continuity; 48% of respondents; 50% sustaining, 43% opt-out). Respondents described *miscommunication* between psychiatry and PCPs: “But if psychiatrist thinks that the medication needs to be increased, and then has to tell [the] physician this message, [then this exchange] can sometimes be odd. The interaction between psychiatrist and physicians should be improved.” (Sustaining DCM). One sustaining DCM also noted, “Doctors are uncomfortable either because they might get more work to do” (*PCP engagement*). In addition, hiring and maintaining fully committed DCMs and psychiatrists for a primary care initiative often proved difficult (*provider continuity*): “[we] supervise the social workers more than psychiatrists due to turnover of the psychiatrists (have had 6 psychiatrists over 3 years)” (Sustaining PCP). Resident turnover also made program fidelity difficult (10% of respondents specifically noted resident **engagement** or **training/knowledge** as barriers).

**External environment** (39% of respondents; 38% sustaining and 43% opt-out) also appeared to affect sustainability. Participants mentioned *competing primary care initiatives*, such as Accountable Care Organizations (ACOs), which often had their own metrics that either added to providers’ workload or did not align with OMH’s required metrics. *Other external factors* included the *mental health infrastructure* as a whole: “the sickest patients psychiatrically have to be referred out. That coordination is tough as it is difficult to collaborate with mental health providers outside of the clinic.” (Sustaining PCP) and *restrictive enrollment requirements* (i.e., clinics could only receive the $150 per-member per-month supplemental payment for Medicaid patients only): “Patients who do not have Medicaid are still screened, so they get “light touches” with the DCM to help get them referred into therapy” (Sustaining Admin).

**Workflow logistics** (32% of respondents; 33% sustaining and 29% opt-out) barriers related to *complicated screening and referral* and *triaging*, which included conducting confidential screening, alerting physicians to depression scores, completing a warm handoff, and convincing patients to enroll. Sustaining interviewees (33%) noted difficulty with screening fatigue while opt-out clinics (29%) reported difficulties around consistency of CC delivery “[the] MA [medical assistants] did PHQ2 but hard to make sure to alert the doctor about PHQ-2 and to do the PHQ-9 if positive” (Opt-out PCP).

**Funding** (30% of respondents; 29% sustaining and 17% opt-out). Interviewees (mostly administrators) remarked on *complex funding streams*. For sustaining clinics, to collect the 25% “retainage” (fee-for-quality), payment practices had to implement a system of quality metrics, which required time and resources. In addition, participants described difficulty using OMH/DOH specific rate codes for CC, not recognized by some electronic health records (EHRs) and often submitted manually. Nonetheless, interview participants noted that this OMH/DOH sponsored program proved essential given NYS restrictions that prohibit social workers from billing for most patients. Opt-out sites cited complex billing infrastructure: “for patients with commercial insurance, each insurance has a different requirement/payment structure” (Opt-out Admin) as well as insufficient numbers of Medicaid patients to make opting into the Medicaid reimbursement program appealing. There were also concerns about *insufficient funding* for all program components: “Billing is not enough, but it’s close. If CM’s have between 70-80 patients that they bill for consistently might break even. Cannot bill [sustainably] for everyone. Doesn’t cover psychiatry/PCP coordinator” (Sustaining PCP).

With the training and accountability provided by this initiative, fewer individuals remarked on barriers related to **information technology/registry** (IT, specifically registry management, screening) (26%), **training/knowledge** (19%), or **buy-in**/**implementation readiness** (10%) (Fig. 2).

#### Facilitators to CC implementation in the sustainability phase

**Fig. 3:**
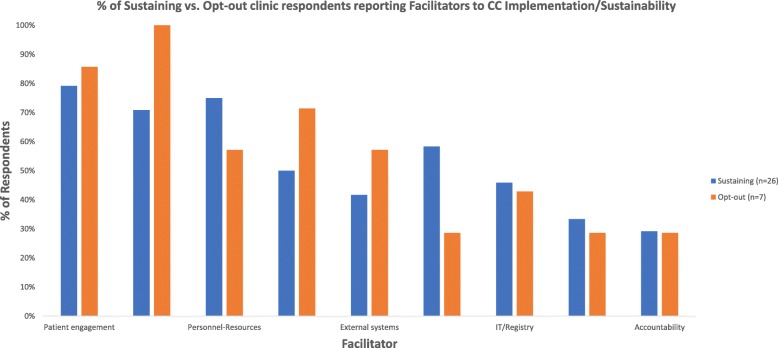
% of Sustaining vs. Opt-out clinic respondents reporting Facilitators to CC Implementation/Sustainability

**Patient engagement** (81% of respondents; 79% sustaining and 86% opt-out) was the most cited facilitator, specifically the use of *personalization/education/motivation* (i.e., message framing, patient preference driven treatment, motivational interviewing, success stories, targeted/tailored psychoeducation). Respondents also emphasized the importance of *warm handoffs*, i.e., PCPs’ real-time introduction of the patient to DCMs: “[When you perform] warm handoffs, then many more [patients] follow up, maybe 80%” (DCM). Respondents also recommended finding *DCMs proficient in engagement*, *reminder systems*, and *appointment flexibility* (Table 5, Fig. 3).

**Table 5:** Facilitators to Collaborative Care implementation/sustainability 1–2 years after a 2 year-implementation program among clinics opting in vs. out of a Medicaid reimbursement sustainability initiative

Facilitators	Total (%)	Sustaining (%)	Opt-out (%)	Quotes
Patient engagement	81	79	86	
Appointment flexibility	26	29	14	
DCM proficiency in engagement	35	29	57	“[She is] very accessible, great clinician…Engaging person which is necessary. She reaches out to the physicians and residents, and they know she is very available except when in session. People knock on door for emergencies.” (Sustaining Admin)
Personalization/education/motivational messaging	48	46	57	“[Message] framing so that avoid stigma” (Opt-in PCP); “Staff matches patients culturally and DCMs are all bilingual in Spanish.” (Opt-in Admin); Engage: Gave gift card/ metro card, health first, talk about depression in positive way, e.g. many will not say depressed, feeling sad” (Opt-out Admin); “Newsletter to talk about positive stories” (Opt-in PCP)
Reminder system	13	17	0	
Warm handoffs	39	42	29	“[When you perform] warm handoffs, then many more [patients] follow up, maybe 80%” (Opt-in DCM)
Provider/staff engagement	77	71	100	
Provider/staff communication	58	54	71	“Mini-teams of Patient Care Administrator + Registered Nurse + PCP will help increase personal accountability for patients.” (Opt-in Admin)
Engage staff	19	13	43	“Involve ALL of the staff, including support staff (nurses, MAs, clerical staff) because it is a culture transformation.” (Opt-out Admin)
Engage PCPs	35	38	29	“Scripting to the PCPs that this will help your panel look better.” (Opt-in DCM) “PCPs join interdisciplinary meeting with psychiatrist and DCM. Scheduled at 1 pm during shift from morning to afternoon and food is provided.” (Opt-in Admin)
Optimize use of psychiatry	26	29	14	“Psychiatrist: 50% face to face visits, 50% for chart reviews, case supervision with team, PCPs come to meeting that psychiatrist. Psychiatrists fill with 3–4 month waiting list quickly, so reserving time for not face-to face allows for more population health model. The psychiatrist sees patient only 1–3 times max.” (Opt-in Admin)
Personnel resources	71	75	57	
Add personnel resources	52	50	57	“We are looking for a SW but unable to find one. Salary offered is very low and the other people not qualified and thinking about increasing the level.” (Opt-in Admin); “The DCM should be co-located in the clinic.” (Opt-in Psych); “More staff: another DCM, more MAs, practice manager…More time with psychiatrist would be helpful.” (Opt-out DCM)
Paraprofessionals	35	42	14	“The data manager [paraprofessional] sends referrals to the DCM and administrator, scheduled appointments, calls patients as reminders, and adds patients to the list for billing purposes.” (Opt-in Admin) “Patient educator allows SW to practice at top of license: appointment reminders, check in on treatment care goals, scheduling, in between DCM appointment contact. Makes sure patient fills new prescription, takes meds. If they have questions, helps monitor the registry” (Opt-in Admin)
Training	55	50	71	
Billing training	6	8	0	
Ongoing training	52	46	71	“A coach teaches care managers engagement [strategies] and [staff] how to get an accurate PHQ score… [there is also] training in motivational interviewing… quarterly training, certification is intensive” (Opt-in DCM). “When you have new residents or attendings, they need to be trained.” (Opt-in Admin)
Screening/referral	52	58	29	
Screening/referral logistics	6	4	14	
Flexibility/QI	6	4	14	
Standardization	45	50	29	“Psych Nurse Practioners (NPs)/Physician Assistants (PAs) came to teach MAs how to do a PHQ-2,-9, which really helped. After the training a competency was developed, and the MAs were evaluated…This was very helping in improving screening rate and quality.” (Opt-in Admin) “Developed a protocol for medication management for depression which is VERY prescriptive. Start sertraline on certain schedule, then add bupropion, etc. 90% of the time that people do not achieve remission is because they have not followed the protocol.’ (Opt-in Admin)
External factors	45	42	57	
Healthcare system	19	17	29	“A lot of systems do not credential SW’s to bill. So coming up with a more streamlined approach to SW billing for hospital systems so that they have an incentive to do it.” (Opt-in PCP)
Leadership commitment	23	25	14	
Optimize long-term/community mental health	13	13	14	“More services would make this program better, specifically group therapy and substance abuse treatment.” (Opt-in Psych)
Leverage national/primary care initiatives	3	4	0	“This model of care fits with clinic’s Patient Centered Medical Home (PCMH) activities which was helpful.” (Opt-in Admin)
Information technology (IT)	45	46	43	
Optimize EHR/registry	16	8	43	“If the EHR could feed into the registry, which would eliminate redundant data entry.” (Opt-in Admin)
Dashboard/mobile technology	10	8	14	“We are putting PHQ-9 on iPads. Idea is to have patient fill out PHQ9. If patient screens positive, will pop up on PCP screen. If question 9 is positive [suicide question], then a hard stop will come up requiring risk assessment.”
IT consultant	10	8	14	“IT person is mandatory for data collection, data analysis, and EHR updates with alerts or other changes to both be user friendly and meet the needs.” (Opt-out Admin)
Telemedicine/psychiatry	23	25	14	“Psychiatric e-consults: If PCP has a question that involves psychiatric med management (NOT a diagnostic question); they use EPIC to send a message to the care manager. Each week they sit down with the psychiatrist for 10-12 min/consult to review chart and make multiple recommendations. These are primarily bipolar disorder or more complex patients, which has reduced face to face encounters by >50%.” (Opt-in Admin)
Funding	32	33	29	
Increase funding	6	4	14	
Leverage current funding streams	26	29	14	“DOH PCMH Patient demonstration project: grant obtained in late 2012 – this helped roll out PCMH model and hire part time LCSW.”
Accountability	29	29	29	“Tracking data at provider level - what site or SW has best patient engagement?” (Opt-in Admin) “Follow up with provider and a lot of support to make sure that every patient screened who is referred.” (Opt-in DCM) “Accountable to program using data and transparently showing data to the entire team” (Opt-in Admin)

*Admin* clinic administrator, *DCM* depression care manager, *Psych* clinic CC psychiatrist, *PCP* primary care provider/champion

**Provider/staff engagement** (77% of respondents; 71% sustaining, 100% opt-out). Participants recommended improving *provider/staff communication* by creating mini inter-disciplinary teams, *engaging staff* *and* *PCPs:* (e.g., “Scripting to the PCPs that this will help your panel look better.”—Sustaining DCM) and *optimizing use of psychiatry*: “50% face to face visits, 50% for chart reviews, case supervision with team…” (Sustaining Admin).

**Personnel resources** (71% of respondents; 75% sustaining, 57% opt-out). Most clinics noted the need for either additional or replacement DCMs and psychiatrists 1–2 years into the sustainability period. Respondents, particularly sustaining clinics recommended hiring paraprofessionals to complete administrative tasks (67% sustaining vs. 0% opt-out): “[Our] patient educator [i.e., paraprofessional] allows the social worker to practice at [the] top of [her] license: [she can make] appointment reminders, check in on treatment care goals, scheduling, in between CM appointment contact… [She also] makes sure [the] patient fills new prescriptions [and] takes meds... [she also] helps monitor the registry” (Sustaining Admin).

**Screening/referral** (52% of respondents; 58% sustaining, 29% opt-out). Respondents recommended *flexibility/quality improvement* initiatives and *standardization*: Successful sustaining sites developed protocols for medication management to help providers at the time of referral: “[We] developed a protocol for medication management for depression, which is VERY prescriptive,…90% of the time that people do not achieve remission is because they have not followed the protocol carefully” (Sustaining Admin).

**Training** (55% of respondents; 50% sustaining, 71% opt-out). Participants emphasized the need for *ongoing training* “A coach teaches care managers engagement [strategies] and [staff] how to get an accurate PHQ score… [there is also] training in motivational interviewing… quarterly training…certification is intensive” (Sustaining DCM). Respondents recommended incorporating training into workflow/schedules of staff, residents, attendings, and care managers: “Involve ALL of the staff, including support staff (nurses, MAs, clerical staff) because it is a culture transformation…Repetition was also very important” (Opt-out Admin).

**IT/Registry** (45% of respondents; 46% sustaining and 43% opt-out): Respondents emphasized incorporating IT staff into the model: “IT person is mandatory for data collection, data analysis, and EHR updates with alerts or other changes to both be user friendly and meet the needs.” (Opt-out Admin). Other facilitators included leveraging *telemedicine/psychiatry e-consults*, internet-based *dashboards/mobile technology* for screening and teaching and *optimizing EHR* for referral, metric tracking and *automating registry* entry: “If PCP has a question that involves psychiatric med management (NOT a diagnostic question), they use EPIC to send a message to the care manager. Each week they sit down with the psychiatrist for 10-12min/consult to review chart and make multiple recommendations. These are primarily bipolar disorder or more complex patients, which has reduced face to face encounters by >50%.” (Sustaining Admin).

**External factors** (45% of respondents; 42% sustaining and 57% opt-out). Respondents felt the *healthcare system’s reimbursement infrastructure* needed improvement as a whole: **“**A lot of systems don’t credential SW’s to bill. So coming up with a more streamlined approach to SW billing for hospital systems so that they have an incentive to do it.” (Sustaining PCP). In addition, *leadership commitment*, *optimizing long-term/community mental health options*, and *leveraging national/primary care initiatives* were other important external factors, particularly for opt-out clinics.

**Funding** (32% of respondents; 33% sustaining and 29% opt-out). Respondents recommended *leveraging current funding streams*, remarking that the initial 2-year CC implementation program was integral to “roll[ing] out PCMH model and hir[ing] a part-time LCSW.” (Opt-out Admin). The current OMH/DOH program reimburses for Medicaid participants to meet billing limitations for Medicaid services provided by social workers. Sustaining clinics in particular noted that this initiative helped them sustain the program: “All funding is coming through OMH per member per month program. If care manager has caseload of at least 50 patients they can cover the program. We think this works so much so that we have applied for a 3rd Care manager.” (Sustaining Admin). Respondents also emphasized *increased funding* to hire more care managers and staff (Fig. 3 ).

**Accountability** (29% for all). One theme that appeared to be a facilitator for multiple other themes (i.e., provider/staff engagement, patient engagement, training, screening/referral) was around creating *accountability* though audit and feedback: *“*Transparency/Accountability [are key]: [We] share the data with the staff without names. They try to make it a little fun/mildly competitive but only once people had bought in. Physicians asked for it [the data] anyway because they were invested in it” (Opt-out Admin).

## Discussion

Training programs with financial incentives improve sort-term CC implementation fidelity, as demonstrated by initiatives like the DIAMOND program [17] and the NYS OMH/DOH CC Initiative [33]. We add to the literature by describing CC processes that promote and inhibit implementation fidelity up to 5 years after initial implementation in a sample of 32 primary care clinics with academic affiliations (defined as serving as a training site for least 1 resident). We found that structural rules (e.g., payment reform) enacted by NYS encouraged most (81%) but not all primary care clinics to continue beyond the CC implementation phase into the sustainability phase. Clinics that saw early clinical improvements and who invested in staffing FTEs were more likely to elect to enter the sustainability phase. Sustaining clinics went on to see stable depression screening rates and treatment titration rates, percentage of patients screening positive for depression, improved DCM contacts/patient and remission rates, and decreased CC enrollment rates/FTE 1 and 2 years into the sustainability phase. Sustaining clinics also maintained high CC fidelity, while clinics that opted-out clinics reported fewer psychiatry and care manager FTEs and patient caseloads/DCM FTE, less psychiatry and care manager time spent in direct patient care, and a lack of PCP champions. Respondents from both sustaining and opt-out clinics revealed that patient and provider engagement and care manager resources were critical factors to ensuring sustainability. Respondents mentioned barriers cited in prior implementation efforts, such as funding, training, workflow logistics, and leadership support factors, less often.

Payment reform alone was not enough to encourage all clinics to continue to provide CC. Respondents surveyed noted that implementation required considerable buy-in from staff and administration, time and resources. In fact, most sites interviewed used multiple funding streams to support CC. Opt-out clinics were often smaller than sustaining clinics and may have lacked the staffing flexibility (e.g., DCM FTEs) to assign staff part-time where needed to make the program successful. Opt-out sites also frequently reported a lack of PCP champions and engaged psychiatrists during the sustainability phase, both shown to correlate with patient activation and remission rates in CC. Relatedly, opt-out clinics tended to be those that did not see early clinical benefits for clients, and hence may not have seen value in continuing the program.

Sustaining and opt-out clinics reported similar barriers, such as lack of resources (e.g., care managers, time) and poor patient and provider engagement in the sustainability phase. Our results are supported by another qualitative analysis of a depression and diabetes CC sustainability program, which identified patient medication concerns, provider concerns around psychotherapy, workloads of staff, and resource barriers [34]. These barriers may accrue over time and become particularly challenging as initial champions of the program turn over. Interest in the program and adherence to key engagement interventions like warm handoffs (only half of site reported routine use) may decline over time. This implementation drift may undermine the very activities that are necessary to generate the visits and associated billings needed to sustain the program.

Respondents identified several factors critical to addressing these barriers to sustaining CC. Patient engagement may respond to behavioral interventions, such as message framing and motivational interviewing ideally delivered via warm handoffs. Ongoing training and accountability [35] (i.e., feedback to providers about their own CC fidelity metrics or patient outcomes) may target provider engagement. Meanwhile, leveraging e-consults may improve psychiatry involvement. Restricted fiscal incentives (applied only to clients with Medicaid) on the other hand may have contributed to some clinics’ decisions to opt-out. Finally, to address resource barriers, many of the sustaining clinics invested in lower-cost administrative assistants to free DCMs to engage in services that are more billable. These assistants also often performed warm handoffs. Our data cannot speak to whether having non-clinical staff perform this role helped or hindered engagement. However, research shows that task shifting may be associated with suboptimal long-term engagement rates and outcomes in mental health [36]. Warm handoffs between PCPs and DCMs correlate with improved remission rates in CC [27], and future research is needed to elucidate how best to incorporate paraprofessionals into the model.

Finally, the focus on healthcare systems with academic affiliations limits the generalizability of these findings. Healthcare systems where residents are trained may have resources not available to nonacademic institutions [22, 24]. Still, many clinics do participate in resident training, and the current sample of clinics is diverse with respect to size and ownership (the majority were public health systems or were community settings with academic affiliations), adding to the generalizability of our findings. In addition, only 10% of all respondents mentioned barriers specifically related to residents.

### Limitations

There were several limitations in this study. We were only able to analyze long-term metrics in 26 of the 32 clinics due to missing data (i.e., opt-out clinics stopped collecting/reporting metrics), relied on descriptive statistics, and were unable to compare metrics to previously published early implementation period metrics due to a change in how we defined metrics. The sustainability intervention was not randomized, further limiting conclusions on effectiveness. However, our mixed methods approach allowed for a granular exploration of clinics successful and unsuccessful in sustaining CC. While we used a purposive approach for qualitative analyses to be representative of all 32 clinics in the initiative and stopped due to saturation, it is possible that we did not fully capture variations, barriers and facilitators across all clinics and furthermore, that participants conflated implementation and sustainability factors. We were underpowered to differentiate the number of barriers and facilitators mentioned by sustaining vs. opt-out sites, and did not employ process evaluation frameworks to better elucidate contextual factors. Finally, primary care clinics affiliated with academic systems limited the generalizability of our results, though they were diverse in size and ownership. The sample size, however, limited analysis of differences by ownership. Nonetheless, this study adds a rarely seen granular view of a large sustainability initiative.

## Conclusion

OMH demonstrated its ability to aid clinics in meeting key domains of integrated care and advancing along the integrated care continuum [37]. Our findings suggest that measurement-based reimbursement programs are a successful strategy for sustaining CC in most but not all clinics. Clinics that saw early clinical improvement and invested in staffing FTEs were more likely to elect to enter the sustainability phase. While limited to settings where residents are trained, our results suggest that successful sustaining of CC may hinge on supporting practices that promote both patient and provider engagement and adequate care manager resources.

## Additional file

Additional file 1: Interview guide: Entire interview guide. (PDF 96 kb)

## Abbreviations

CC: Collaborative care (integration of primary care and mental health care programs)
DCM: Depression care manager (often a social worker trained in providing therapy and integrated in the primary care setting)
DOH: Department of Health (New York State’s Department of Health)
FTE: Full-time equivalent (hours worked by one employee on a full-time basis)
OMH: Office of Mental Health (New York State’s agency for mental health)
PCP: Primary care physician (the primary medical provider for a patient in the primary care setting)
PHQ: Patient Health Questionnaire (validated depression screening)
