# Supplementary material for: Sustainability of collaborative care management for depression in primary care settings with academic affiliations across New York State
Source: Implement Sci. 2018 Oct 12;13:128. doi: 10.1186/s13012-018-0818-6 (PMC6186053; doi:10.1186/s13012-018-0818-6)
Supplement: Supplementary file 1 — Interview guide: Entire interview guide. (PDF 96 kb) [file 13012_2018_818_MOESM1_ESM.pdf]

Additional File 1. Interview Guide

SITE VISIT QUESTIONS

Basic Questions
- **Size of practice**
- **Demographics of patients**
- **% of Medicaid Patients**
- **Number of primary medical doctors (PMDs) (individuals and Full Time Equivalent (FTE))**
- **Number of Psychiatrists (individuals and FTE)**
- **Number of depression care managers (individuals and FTE)**
- **What is the training and licensure of the care manager? (social work, psychology, etc?)**
- **Number of care managers (individuals and FTE) who are clinicians (e.g., social workers), number of non-clinician care managers (individuals and FTE)**
- **Email address(es) to add to *potential* listserv to share ideas on collaborative care:**

Administrator
- What are the funding streams for this program? How healthy is the program financially? What are the biggest fiscal challenges? How does the clinic afford the extra operational costs?

- What are your thoughts about the security of financing and fiscal sustainability of this model? What percentage of the costs of this program are you able to cover?

- Is the care manager on-site? How accessible is he or she? How many times in the past month did you knock on his/her or door or stop by his/her office?

- In what ways is leadership committed to the project? How could leadership be more supportive?

- What makes this program challenging? What would make this program even better?

- Do you have any best practices to share? What might you like to learn from other such programs?

- In general, to what degree do you believe that the collaborative care approach is effective in improving outcomes in patients with depression? (1) Not effective, (2) Minimally effective, (3) Somewhat effective, (4) Very effective, (5) Extremely effective

Psychiatrist

- How much time do you have at the clinic and how do you divide your time: # of hours, hours for patient care, hours for care manager supervision, dedicated time with PMDs?
- How many patients are in the program? How many patients on your list?
- Do you look at the registry? How many times did you review the registry in the last month? What do you use it for?
- Is the care manager on-site? How accessible is he or she? How many times in the past month did you knock on his/her or door or stop by his/her office?
- How do you connect with PMDs? Do they contact you in between sessions, and how long does it take you to get back to them?
- How many times did PMDs and care manager contact you in the last month? Do they only contact you when you are physically there or also by phone/pager at other times during the week?
- Do you have a sense of what makes PMDs refer more or less?
- What percent of the PMDs refer to SW and/or psychiatrist? What are the referral rates across the PMDs?
- What percent of depressed patients in the clinic show at least some improvement? What percent recover? How can you tell who is not improving? What do you do about those that are not getting better?
- Do you have a protocol for suicidal risk? How many times has it been used in last 1 year?
- What makes this program challenging?
- What would make this program even better?
- Do you have any best practices to share? What might you like to learn from other such programs?
- What medications do the PMDs most commonly prescribe?
- What medications do PMDs prefer to be prescribed to a psychiatrist?
- What kind of in-services or educational sessions have you done in the clinic? How many have you done in the past year?
- In general, to what degree do you believe that the collaborative care approach is effective in improving outcomes in patients with depression? (1) Not effective, (2) Minimally effective, (3) Somewhat effective, (4) Very effective, (5) Extremely effective

PMD

- Who are the PMD champions?
- How do they work on this project?
- How do you decide whom to refer to the care manager versus the psychiatrist?
- For what reasons and how many times did you refer patients in the past month to the care manager? To the Psychiatrist? What percent of the PMDs refer to the care manager and/or psychiatrist? What are the referral rates across the PMDs? Is there variation?
- Is the care manager on-site? How accessible is he or she? How many times in the past month did you knock on his/her or door or stop by his/her office?
- The recommended components of the collaborative care model are to screen everyone with a PHQ2 followed by a PHQ9 followed by patient preference driven care and referral to a behavioral care manager and who then works in concert with the primary care physician.
- How do you engage patients in the collaborative care process?
- What makes this program challenging?
- What would make this program even better? What might you like to learn from other such programs?
- Do you have any best practices to share?
- In what ways is leadership committed to the project? How could leadership be more supportive?
- How do you communicate with the psychiatrist?
- How many times in last month have you contacted the psychiatrist outside of hours psychiatrist is physically present? How long did it take for him/her to get back to you?
- How many active open cases do you and other PMDs have in the program on average?
- What medications do you most commonly prescribe?
- Do you prescribe controlled substances for psychiatric conditions regularly?
- What medications would you prefer to be prescribed to a psychiatrist?
- In general, to what degree do you believe that the collaborative care approach is effective in improving outcomes in patients with depression? (1) Not effective, (2) Minimally effective, (3) Somewhat effective, (4) Very effective, (5) Extremely effective

- Are there any strategies that you have found or heard were successful in engaging patients to participate or take their medications?

Care Manager

- Full vs part time? # hours on site per week? What other jobs do you have at this clinic? How many open, active patients do you have?
- Physical setting: Onsite office? Open-door policy, are you visible in the clinic?
- How is your role defined?
- How is your day spent? Do you spend part of your day on duties outside of this program?
- How much time do you spend on care coordination with team vs. registry vs. patient contacts?
- How do you use phone vs. in person vs. email/texts to engage patients?
- Is there a standing, regular interdisciplinary team meeting? Who attends and how often?
- The recommended components of the collaborative care model are to screen everyone with a PHQ2 followed by a PHQ9 followed by patient preference driven care and referral to a behavioral care manager and who then works in concert with the primary care physician. How do you engage patients in the collaborative care process?
- How do the screenings get done, scored, and evaluated? Do you have a flow/process diagram or use the EHR?
- How do you engage patients who screen positive to enroll in the program?
- What are your admission and discharge criteria? Is the population well-defined?
- What have been your greatest challenges?
- What have been your greatest successes in this? Do you have any best practices to share?
- What would make this program even better? What might you like to learn from other such programs?
- Where do you get referrals (% from PMDs, % from screen, other)?
- What are the most common dispositions? (keep patient, discharge from program, referral out, other?)
- How do you do hand-offs? (% face to face, via notes in chart, phone, etc)
- Registry: Do you use AIMS, Excel, or other? Does the registry and tracking of data help? How so?
- Have you had any completed suicides since the program started? Any suicide attempts?
- Do you have a protocol for suicidal risk? How many times has it been used in last 1 year?

- In what ways is leadership committed to the project? How could leadership be more supportive?

- How many PMDs support the program and refer patients to it? What percent? (0-20%, 21-40%, etc)

- In what ways is the care provided accountable?

- How often do you meet with the psychiatrist for case review? What is it like? What would make it more helpful?

- How do you select which cases to review?

- How many times did you contact the psychiatrist in between scheduled sessions in the past month? How long did it take for him/her to get back to you?

- Resources: Other social worker available for issues related to housing, disability, HHA, etc

- Does anyone help you schedule follow-ups or coordinate care (i.e., patient navigator/extender)?

- In general, to what degree do you believe that the collaborative care approach is effective in improving outcomes in patients with depression? (1) Not effective, (2) Minimally effective, (3) Somewhat effective, (4) Very effective, (5) Extremely effective

- How confident are you that you can ensure that your depressed patients take their antidepressants and/or attend their behavioral care/psychiatry visits after diagnosis? (1) Not confident at all, (2) Minimally confident, (3) Somewhat confident, (4) Very confident, (5) Extremely confident.
